# Predictors of Clinically Driven Target Lesion Revascularization After Endovascular Treatment of Femoropopliteal Chronic Total Occlusions

**DOI:** 10.3390/jcm15197438

**Published:** 2026-09-24

**Authors:** Busra Guvendi Sengor, Barkın Kultursay, Mehmet Sengor, Mustafa Ferhat Keten, Tuba Unkun, Abdulrahman Naser, Ender Ozgun Cakmak, Regayip Zehir

**Affiliations:** 1 Department of Cardiology, Kartal Kosuyolu Education and Research Hospital, Istanbul 34865, Turkey; ferhat_keten@hotmail.com (M.F.K.); tubaunkun@yahoo.com (T.U.); zehirregayip@yahoo.com (R.Z.); 2 Department of Cardiology, Tunceli State Hospital, Tunceli 62100, Turkey; bkultursay@gmail.com; 3 Department of Cardiovascular Surgery, Bezmialem Vakıf University Hospital, Istanbul 34093, Turkey; sengor_mehmet@hotmail.com; 4 Department of Cardiology, Kırklareli Education and Research Hospital, Kırklareli 39010, Turkey; abdulrahman_naser@hotmail.com; 5 Department of Cardiology, Istanbul Health and Technology University, Istanbul 34445, Turkey; enderozguncakmak@gmail.com

**Keywords:** femoropopliteal disease, chronic total occlusion, target lesion revascularization

## Abstract

**Background:** Clinically driven target lesion revascularization (CD-TLR) following endovascular recanalization of femoropopliteal chronic total occlusions (CTOs) remains a primary determinant of long-term procedural durability. This study aimed to identify clinical, anatomical, and procedural predictors associated with 12-month CD-TLR following successful femoropopliteal CTO endovascular treatment. **Methods:** This retrospective single-center cohort study evaluated 246 successfully recanalized patients who underwent endovascular treatment for femoropopliteal CTO between June 2020 and June 2025. The primary endpoint was 12-month CD-TLR. To overcome severe multicollinearity and prevent overfitting among correlated predictors, five survival algorithms were benchmarked across the complete 27-variable candidate set. Model discrimination was evaluated using Harrell’s concordance index (C-index) with bootstrap correction. **Results:** Over a 12-month time-to-event follow-up (median 365 days), CD-TLR occurred in 114 patients (46.3%). Multivariable-regularized Elastic Net Cox regression retained 22 informative predictors, achieving an apparent C-index of 0.715 (95% CI: 0.66–0.77) and an optimism-corrected C-index of 0.653. Higher occluded below-the-knee vessel count, baseline Rutherford classes 4–6, popliteal CTO extension, distal runoff impairment, active smoking, and overall concomitant interventions showed positive associations with 12-month CD-TLR risk. Conversely, shorter lesion length (<5 cm) and female sex showed inverse associations, while findings related to stent dimensions were considered exploratory. **Conclusion:** Twelve-month CD-TLR after endovascular treatment of femoropopliteal CTO was associated with greater distal vascular disease burden, anatomical complexity, a worse baseline Rutherford class and impaired distal runoff. The Elastic Net model provided the highest optimism-corrected discrimination among the evaluated strategies, although prospective external validation remains necessary before broad clinical application.

## 1. Introduction

Peripheral artery disease remains a major cause of morbidity, functional impairment, and limb-related adverse events worldwide [1,2,3]. The superficial femoral artery (SFA) chronic total occlusions (CTOs) represent a challenging subset because of their greater lesion complexity, longer occlusion length, calcification burden, and frequent extension into the popliteal segment [4,5]. These anatomical characteristics may complicate lesion crossing and influence both procedural strategy and subsequent durability of revascularization. Although advances in endovascular techniques and device technology have substantially expanded the feasibility of revascularization, loss of primary patency and restenosis remain important limitations after treatment of complex SFA CTO lesions.

Restenosis after femoropopliteal endovascular intervention is multifactorial and may be influenced by clinical characteristics, lesion morphology, distal runoff, calcification, procedural strategy, and device-related factors. Previous studies have identified several individual predictors of restenosis and target lesion failure; however, the relative contribution of closely interrelated anatomical and procedural variables remains difficult to determine [6,7,8]. This issue is particularly relevant in patients with CTOs, in whom lesion length, calcification severity, distal vessel involvement, crossing strategy, stent characteristics, and concomitant interventions may coexist and interact.

Beyond anatomical patency, the need for repeat intervention represents an important measure of the long-term durability of femoropopliteal endovascular treatment. Clinically driven target lesion revascularization (TLR) is a particularly relevant outcome, as it captures the need for repeat intervention at the previously treated segment. Understanding the factors associated with TLR may therefore provide clinically meaningful insight into treatment durability and help identify patients at increased risk of recurrent intervention following CTO recanalization.

Risk assessment after femoropopliteal CTO revascularization is further complicated by the interdependence of anatomical and procedural characteristics. Lesion length, calcification, distal disease extension, runoff status, device dimensions, stent use, and concomitant interventions are not independent treatment attributes but often reflect the same underlying anatomical complexity and operator decision-making. Conventional multivariable regression remains widely used to evaluate predictors of vascular outcomes; however, identifying independent determinants remains challenging in complex lesion subsets due to the interplay of numerous correlated clinical, anatomical, and procedural variables [9,10]. Although several predictors of adverse outcomes following femoropopliteal intervention have previously been described, the combined contribution of clinical characteristics, lesion complexity, distal disease involvement, and procedural factors to clinically driven repeat intervention after CTO recanalization remains incompletely characterized. Accurately determining these risk factors helps clinicians anticipate patency loss, guide post-procedural surveillance, and tailor revascularization modalities to individual patient risk profiles. Accordingly, the present study aimed to evaluate clinical, anatomical, and procedural factors associated with 12-month clinically driven TLR following endovascular treatment of femoropopliteal CTOs and explore their combined prognostic contribution using complementary survival modeling approaches.

## 2. Material and Methods

### 2.1. Study Design and Population

This retrospective, single-center study initially screened 265 patients who underwent endovascular treatment for femoropopliteal CTO between June 2020 and June 2025. Of these, 19 patients were excluded before follow-up cohort assembly, including 7 patients with primary technical recanalization failure and 12 patients because of non-eligibility or incomplete baseline records. The final analyzed cohort therefore comprised 246 patients with successful index femoropopliteal CTO recanalization who were included in the 12-month time-to-event analysis (Figure 1). Femoropopliteal CTO was defined as complete occlusion involving the superficial femoral artery, with or without extension into the popliteal artery. Popliteal CTO and popliteal trifurcation involvement were recorded separately to characterize distal extension and anatomical complexity. The unit of analysis was the individual patient.

A total of 265 patients undergoing endovascular recanalization for femoropopliteal chronic total occlusions (CTOs) were screened from the registry. Nineteen patients were excluded prior to follow-up assembly, including 7 primary technical crossing failures and 12 with non-eligible or incomplete baseline parameters. The final analyzed cohort (*n* = 246) comprised successfully recanalized procedures. The cohort was stratified into patients who experienced 12-month Clinically Driven Target Lesion Revascularization (CD-TLR; *n* = 114, 46.3%) and those remaining free from CD-TLR (*n* = 132, 53.7%).

The study population included patients with and without a history of previous peripheral endovascular intervention. Previous SFA, iliac, and below-the-knee interventions were recorded separately. Baseline demographic, clinical, preprocedural, anatomical and procedural characteristics were collected from the hospital database. Follow-up data were obtained from hospital records and national databases. The study protocol was designed and executed in strict accordance with the STROBE (Strengthening the Reporting of Observational Studies in Epidemiology) guidelines and the TRIPOD (Transparent Reporting of a multivariable prediction model for Individual Prognosis Or Diagnosis) statement. The study protocol was approved by the institutional ethics committee, and the requirement for written informed consent was waived because of the retrospective design. The study was conducted in accordance with the principles of the Declaration of Helsinki.

### 2.2. Preprocedural Assessment

Preprocedural variables included demographic characteristics, cardiovascular risk factors, comorbidities, smoking status, CHA_2_DS_2_-VASc score, previous peripheral arterial interventions, baseline Rutherford classification, ankle–brachial index (ABI), and laboratory parameters. Lesion-related anatomical characteristics included CTO length, popliteal stenosis and CTO, popliteal trifurcation involvement, CTOP and TASC II classifications, PACSS calcification grade, and distal runoff flow grade. ABI was measured before the procedure using Doppler ultrasonography as the ratio of the higher ankle systolic pressure to the higher brachial systolic pressure. Angiographic lesion complexity was assessed using the CTOP and TASC II classifications [11,12]. Calcification severity was graded according to the Peripheral Academic Research Consortium (PACSS) classification, and distal runoff was evaluated using a three-grade angiographic runoff score [13,14].

### 2.3. Procedural Characteristics

Endovascular procedures were performed by teams experienced in peripheral vascular interventions. Treatment strategy was individualized according to lesion anatomy and angiographic characteristics. CTO crossing was performed using either an intraluminal or subintimal approach, with a retrograde approach used when considered necessary.

Following successful lesion crossing, balloon predilatation was performed. Predilatation balloon diameter was selected according to the angiographically estimated reference-vessel diameter. Intravascular imaging was not used for vessel sizing or procedural guidance. Stent implantation was performed in the presence of flow-limiting dissection following balloon angioplasty. When stenting was required, stent diameter was similarly selected according to the angiographically estimated reference-vessel diameter. Self-expanding nitinol stents, SUPERA^TM^ interwoven nitinol stents (Abbott Vascular, Santa Clara, CA, USA), and balloon expandable covered stent grafts were used according to lesion characteristics and procedural requirements.

Procedural variables recorded included crossing strategy, use of a retrograde approach, predilatation balloon diameter, stent implantation and type, number of implanted stents, maximum stent diameter, total stent length, stent overlap, post-dilatation, POBA-only treatment, and concomitant iliac or below-the-knee interventions. Procedure-related dissections were characterized according to their severity, degree of luminal compromise, morphology, and effect on distal flow. Postprocedural ABI was also recorded.

Technical success was defined as successful wire and device crossing of the target femoropopliteal CTO with restoration of brisk antegrade flow, residual diameter stenosis <30% on final angiography, and absence of residual flow-limiting dissection following balloon angioplasty or bailout stenting.

### 2.4. Study Outcomes

Patients were followed for 12 months after the index procedure using institutional medical records and available national health records. The primary endpoint was clinically driven target lesion revascularization (CD-TLR) within 12 months following successful index revascularization. CD-TLR was defined as repeat endovascular revascularization involving the previously treated target lesion, performed for recurrent or worsening limb symptoms or other clinical findings warranting repeat intervention.

Target vessel revascularization (TVR) involving a segment outside the previously treated target lesion was not included in the primary endpoint and was evaluated separately when available. Because protocol-mandated duplex ultrasonography or other systematic vascular imaging surveillance was not performed throughout the cohort, anatomical restenosis in the absence of clinically driven repeat intervention could not be systematically assessed and was therefore not considered the primary endpoint. Additional follow-up outcomes, including amputation, surgical bypass, thrombectomy, in-hospital mortality, and antiplatelet therapy status, were recorded and are presented descriptively. Surgical bypass and thrombectomy involving the previously treated target lesion occurred only in patients who had already undergone endovascular CD-TLR and were therefore not counted as separate CD-TLR events.

### 2.5. Statistical Analysis

Continuous variables were assessed for normality using the Shapiro–Wilk test, with normally distributed data presented as mean ± standard deviation (SD) and non-normally distributed data expressed as median with interquartile range (IQR). Categorical variables are presented as counts and percentages, and baseline clinical, anatomical, and procedural characteristics were compared between patients with and without 12-month CD-TLR using the independent-samples *t*-test, Mann–Whitney U test, Pearson’s chi-square test, or Fisher’s exact test, as appropriate. Missing covariate data were minimal (<1.5%) and handled using Multiple Imputation by Chained Equations (MICE) with 10 imputed datasets under Rubin’s rules. Sensitivity analyses comparing imputed data with complete cases yielded identical results. A comprehensive survival modeling framework was executed incorporating the complete set of 27 candidate variables. To address potential overfitting and multicollinearity among closely correlated predictors, we benchmarked multiple survival modeling strategies, including stepwise Cox regression, least absolute shrinkage and selection operator (LASSO) Cox regression, Ridge Cox regression, Elastic Net Cox regression, and random survival forests (Supplementary Material). Hyperparameters for regularized models were optimized via 10-fold cross-validation. Model discrimination was evaluated using Harrell’s concordance index (C-index) alongside 1000-resample bootstrap optimism correction to quantify internal validity. The final model was selected based on superior discrimination evaluated using Harrell’s concordance index, with adjusted hazard ratios (HRs) and 95% confidence intervals (CIs) calculated accordingly. All statistical tests were two-tailed, and a *p*-value < 0.05 was considered statistically significant, with all analyses executed using R (version 4.3.2; R Foundation for Statistical Computing, Vienna, Austria) with the survival, survminer, glmnet, randomForestSRC, DiagrammeR, and ggplot2 packages.

## 3. Results

### 3.1. Baseline Characteristics

A total of 246 patients undergoing endovascular treatment for femoropopliteal CTO were included, of whom 114 (46.3%) experienced CD-TLR within 12 months, whereas 132 (53.7%) maintained freedom from CD-TLR. Most baseline clinical characteristics were comparable between the CD-TLR and No CD-TLR groups. Among anatomical characteristics, distal runoff flow grade (*p* = 0.01) and the number of occluded below-the-knee vessels (*p* = 0.02) differed significantly between the groups. In contrast, CTOP classification, TASC II classification, CTO length, popliteal CTO involvement, popliteal trifurcation involvement, and PACSS calcification grade did not differ significantly between groups. Most procedural characteristics, including POBA-only treatment, self-expanding and Supera stent implantation, stent count, total stent length, maximum stent diameter, and concomitant interventions, were also comparable between groups. Dissection severity grade was the only procedural characteristic that differed significantly between the groups (*p* = 0.02). Baseline clinical, anatomical, and procedural characteristics are summarized in Table 1. Twelve-month clinical and reintervention outcomes are summarized in Table 2.

### 3.2. Model Comparison and Final Elastic Net Model

To account for multicollinearity and reduce the risk of overfitting among correlated clinical, anatomical, and procedural covariates, all 27 candidate predictors were evaluated across five survival modeling strategies (Supplementary Tables S1 and S2). Random survival forests yielded the highest apparent C-index (0.724); however, its discrimination decreased substantially after bootstrap optimism correction (0.582). Elastic Net Cox regression demonstrated an apparent C-index of 0.715 (95% CI, 0.66–0.77) and the highest optimism-corrected C-index of 0.653 (95% CI, 0.598–0.708). The corresponding optimism-corrected C-indices were 0.645 for LASSO Cox regression, 0.641 for Ridge Cox regression, and 0.635 for stepwise Cox regression. Based on its optimism-corrected discrimination and ability to accommodate correlated predictors, Elastic Net Cox regression was selected as the final modeling framework. The relative importance ranking of predictors derived from the random survival forest model across the complete 27-variable set is illustrated in Figure 2.

Relative variable importance scores (VIMP) illustrating the contribution of all 27 candidate predictors to the non-parametric Random Survival Forest ensemble. Higher VIMP values indicate greater predictive contribution to 12-month CD-TLR following femoropopliteal CTO endovascular intervention, demonstrating strong concordance with the primary regularized Elastic Net Cox regression model.

The final Elastic Net model retained 22 variables. The largest positive coefficients were observed for the number of occluded below-the-knee vessels (β = 0.2666; HR 1.31, 95% CI 1.08–1.62), baseline Rutherford class (β = 0.2633; HR 1.30, 95% CI 1.06–1.60), and impaired distal runoff (β = 0.2136; HR 1.24, 95% CI 1.02–1.51). Additional variables retained with positive coefficients included popliteal CTO involvement, active smoking, and concomitant interventions. Inverse coefficients were observed for shorter CTO length (<5 cm) and female sex, and stent dimensions. However, the findings related to stent dimensions were not supported by the stented-only sensitivity analysis and were therefore considered exploratory. The complete shrinkage coefficients and corresponding hazard ratios are presented in Table 3.

In a sensitivity analysis restricted to patients who underwent stent implantation (*n* = 160), maximum stent diameter, total stent length, stent count, and stent overlap were not significantly associated with 12-month CD-TLR after multivariable adjustment (Supplementary Table S3). The magnitude and direction of the associations retained in the final Elastic Net model are illustrated in Figure 3, while the Kaplan–Meier curve shows freedom from CD-TLR during the 12-month follow-up (Figure 4). The retained variables are further organized by clinical, anatomical, and procedural domains in Figure 5, with individual coefficient profiles provided in Supplementary Figure S1.

Visual representation of adjusted hazard ratios (HRs) and 95% confidence intervals for all 22 predictors retained in the final regularized Elastic Net Cox regression model for 12-month Clinically Driven Target Lesion Revascularization (CD-TLR): Variables with HR > 1.0 (orange markers) indicate positive associations with CD-TLR, whereas variables with HR < 1.0 (green markers) indicate inverse associations.

Cumulative freedom from 12-month CD-TLR across the entire analyzed femoropopliteal CTO cohort (*n* = 246): The solid blue line represents the Kaplan–Meier survival estimate, with shaded regions denoting 95% point-wise confidence intervals. The overall 12-month freedom from CD-TLR was 53.7% (95% CI: 47.9% to 59.5%), corresponding to a cumulative 12-month CD-TLR event rate of 46.3% (*n* = 114). The corresponding numbers of patients at risk at 3-month intervals are displayed below the time axis.

Schematic mapping of the 22 retained variables from the final regularized Elastic Net Cox regression model for 12-month Clinically Driven Target Lesion Revascularization (CD-TLR): The central node represents the 12-month CD-TLR outcome. Left-sided nodes (orange boxes and solid lines) depict variables with positive associations (HR > 1.0) categorized into clinical/laboratory markers, anatomical/lesion burden, and procedural/interventional sub-domains. Right-sided nodes (green boxes and dashed lines) depict variables with inverse associations (HR < 1.0) categorized into anatomical/morphological factors, procedural/device dimensions, and clinical/demographic substrates.

## 4. Discussion

In this retrospective single-center study of 246 patients undergoing endovascular treatment for femoropopliteal CTO, CD-TLR within 12 months occurred in 46.3% of patients. The final Elastic Net Cox model retained 22 variables, with the strongest positive associations observed for greater below-the-knee disease burden, a higher baseline Rutherford class and impaired distal runoff. Other variables, including popliteal CTO involvement, a higher PACSS calcification grade, active smoking, and concomitant interventions, were retained with smaller positive coefficients. Among the five modeling strategies, Elastic Net Cox regression had the highest optimism-corrected C-index, whereas the random survival forest showed a marked decrease in performance after optimism correction.

Our findings are consistent with previous studies highlighting the importance of distal disease burden. In a large multicenter analysis of 1889 patients with femoropopliteal disease, chronic total occlusion and poor below-the-knee runoff independently predicted restenosis across both TASC II class A-C and class D lesions [7]. Similarly, Boc et al. reported that impaired infrapopliteal runoff was associated with reduced primary patency after SFA angioplasty [15]. In our study, popliteal CTO involvement, greater below-the-knee disease burden, and impaired distal runoff were retained in the final Elastic Net model, supporting an association between more extensive distal disease and subsequent CD-TLR. Severe calcification, assessed by the PACSS grade, and active smoking also showed positive associations in the Elastic Net model. Consistent with these findings, a recent intravascular ultrasound study demonstrated that severe calcification was associated with a higher risk of restenosis following femoropopliteal endovascular intervention [16], whereas Baril et al. identified current or former smoking as an independent predictor of primary patency loss after endovascular treatment of TASC II D femoropopliteal lesions [17].

The complex lesion profile of our cohort may have contributed to the high CD-TLR rate. Femoropopliteal CTOs have been associated with a greater need for repeat revascularization than non-CTO lesions [18]. However, the event rate in our cohort was higher than that reported in previous studies, suggesting that CTO anatomy alone may not explain this finding. Differences in lesion characteristics, previous interventions, and treatment strategy may also have contributed [19].

This is particularly relevant in our cohort, in which 87% of patients had undergone previous SFA intervention. Laser atherectomy has been studied in complex peripheral artery disease, including femoropopliteal in-stent restenosis and occlusion [20,21,22]. These studies suggest that lesion type and treatment strategy need to be considered when comparing different femoropopliteal cohorts. In our cohort, laser or other atherectomy, drug-coated balloons, intravascular lithotripsy, and intravascular imaging were not used. Moreover, the available data did not allow reliable distinction between de novo lesions and in-stent restenosis or occlusion. Differences in lesion characteristics and the absence of contemporary adjunctive technologies should be considered when interpreting our findings and comparing them with contemporary endovascular studies.

Several procedural variables showed inverse associations with CD-TLR in the primary model, with predilatation balloon diameter demonstrating the strongest inverse association. Because balloon size was selected according to visually estimated vessel diameter, this association may mainly reflect vessel caliber and lesion anatomy rather than a direct protective effect of larger balloons. Similarly, the associations with larger stent diameter and greater stent number may reflect lesion characteristics, procedural strategy, and operator selection. Because stent-related variables were coded as zero in patients without stent implantation, the observed associations may partly reflect treatment selection rather than independent effects of stent dimensions. In the sensitivity analysis restricted to stented patients, maximum stent diameter, total stent length, stent count, and stent overlap were not significantly associated with CD-TLR. Therefore, these findings should be interpreted cautiously and should not be considered evidence that larger or more extensive stent implantation is protective.

The comparison of modeling strategies provides an additional exploratory aspect of this study. Elastic Net Cox regression showed the highest optimism-corrected discrimination (C-index 0.653), although differences among the regression-based models were modest. Random survival forest had the highest apparent discrimination but showed a marked decrease after optimism correction, suggesting greater susceptibility to overfitting in this relatively small cohort. The performance of Elastic Net may be related to its ability to handle correlated anatomical, clinical, and procedural variables. However, overall model discrimination was modest, and the model has not been externally validated.

From a clinical perspective, our findings suggest that CD-TLR after femoropopliteal CTO recanalization is associated with anatomical complexity, particularly distal disease burden, popliteal CTO involvement, severe calcification, and impaired distal runoff. Considering clinical, anatomical, and procedural characteristics together may provide a more comprehensive assessment of CD-TLR risk than focusing on individual factors alone. The Elastic Net model may be useful in this setting by accounting for correlations among these clinical, anatomical, and procedural characteristics. However, our study does not establish whether closer surveillance or a different endovascular strategy would improve outcomes in these patients. This is particularly important given the retrospective single-center design, moderate model discrimination, lack of external validation, and absence of systematic imaging surveillance. Further prospective studies are needed before these findings can be used to guide follow-up or treatment decisions.

## 5. Limitations

Several limitations should be acknowledged. First, this was a retrospective, single-center study, which may have introduced selection bias and limits the generalizability of our findings. Second, protocol-mandated duplex ultrasonography or other systematic vascular imaging surveillance was not available throughout follow-up. Therefore, the study was designed to evaluate clinically driven target lesion revascularization (CD-TLR) rather than the incidence of asymptomatic anatomical restenosis or primary patency loss, and non-driven lesions not leading to repeat intervention could not be captured. Third, the cohort was characterized by a high prevalence of previous peripheral interventions, including previous SFA intervention in 87% of patients. Detailed information regarding previous SFA treatment, including previous device type, timing of intervention, and systematic differentiation between de novo CTO, in-stent restenosis, and complete in-stent occlusion, was unavailable. This heterogeneity may have influenced both procedural strategy and subsequent risk of CD-TLR. Fourth, contemporary adjunctive technologies, including drug-coated balloons, atherectomy, laser atherectomy, intravascular lithotripsy, and intravascular imaging, were not used in this cohort, which should be considered when extrapolating the findings to contemporary endovascular practice. Quantitative reference-vessel measurements were also not systematically available, precluding reliable assessment of device-to-vessel ratios. Finally, the predictive analyses were derived from a relatively modest single-center cohort and lacked external validation. Accordingly, the modeling results should be regarded as exploratory and require prospective validation in independent populations before clinical application.

## 6. Conclusions

In patients undergoing endovascular treatment of femoropopliteal CTO, the risk of 12-month CD-TLR was primarily associated with greater distal disease burden, anatomical complexity, a worse baseline Rutherford class and impaired distal runoff. Regularized survival modeling provided a useful framework for evaluating correlated clinical, anatomical, and procedural variables. These findings remain hypothesis-generating and require prospective external validation before clinical application.

## Figures and Tables

**Figure 1:**
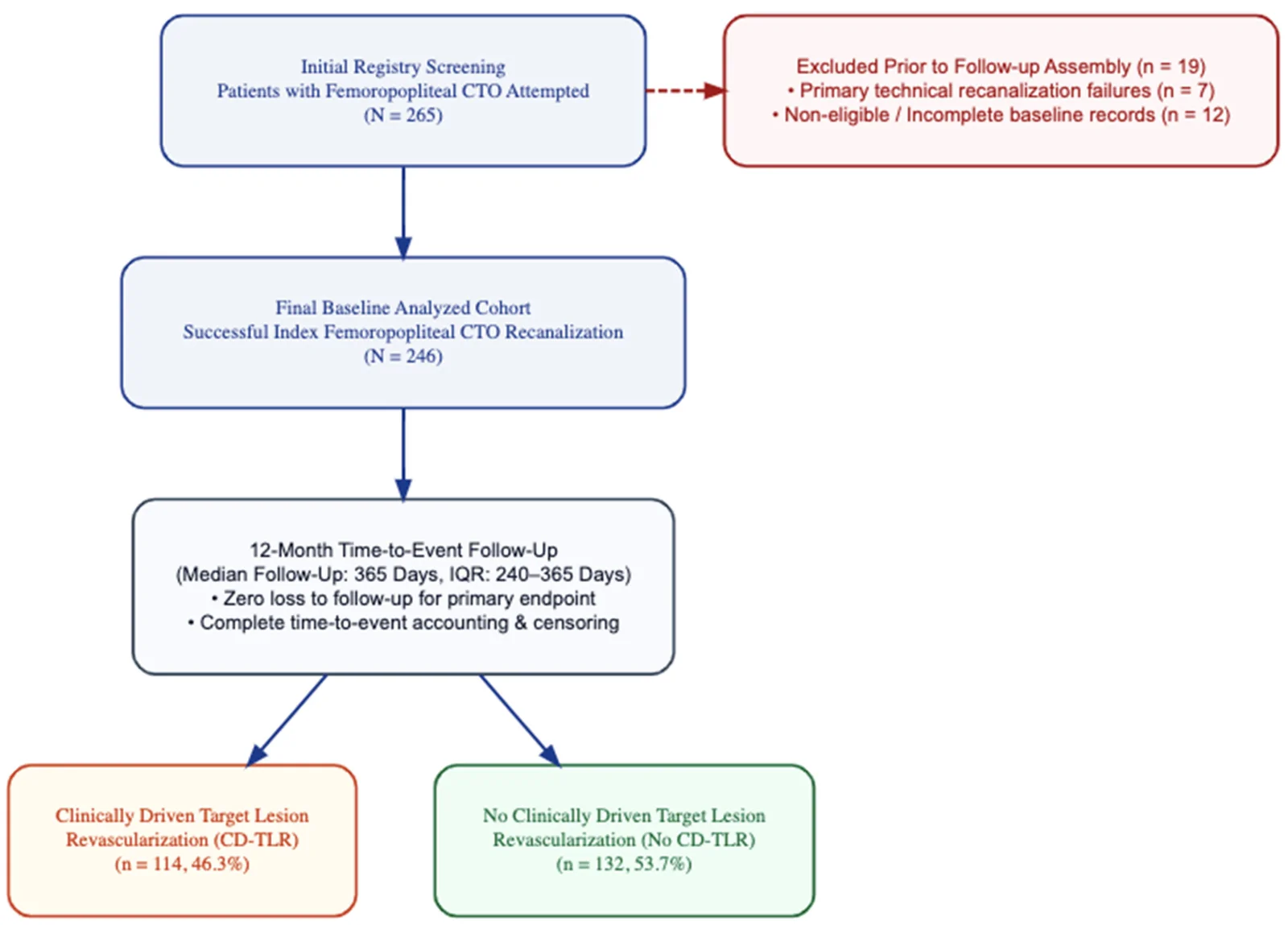
STROBE Flow Diagram of Study Participant Selection, Follow-up, and Outcome Classification.

**Figure 2:**
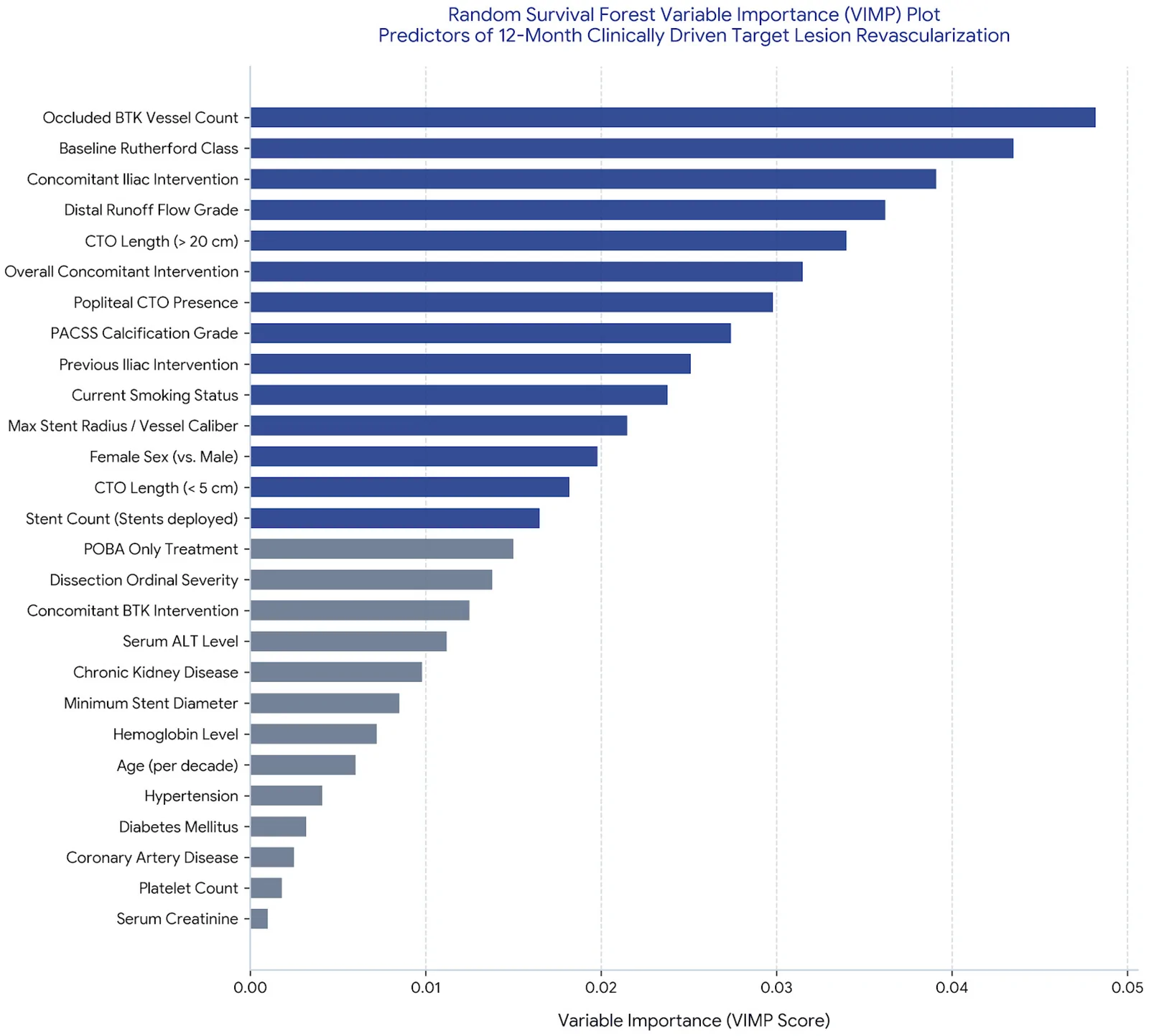
Variable Importance (VIMP) Plot Derived from the Random Survival Forest Model for 12-Month Clinically Driven Target Lesion Revascularization (CD-TLR).

**Figure 3:**
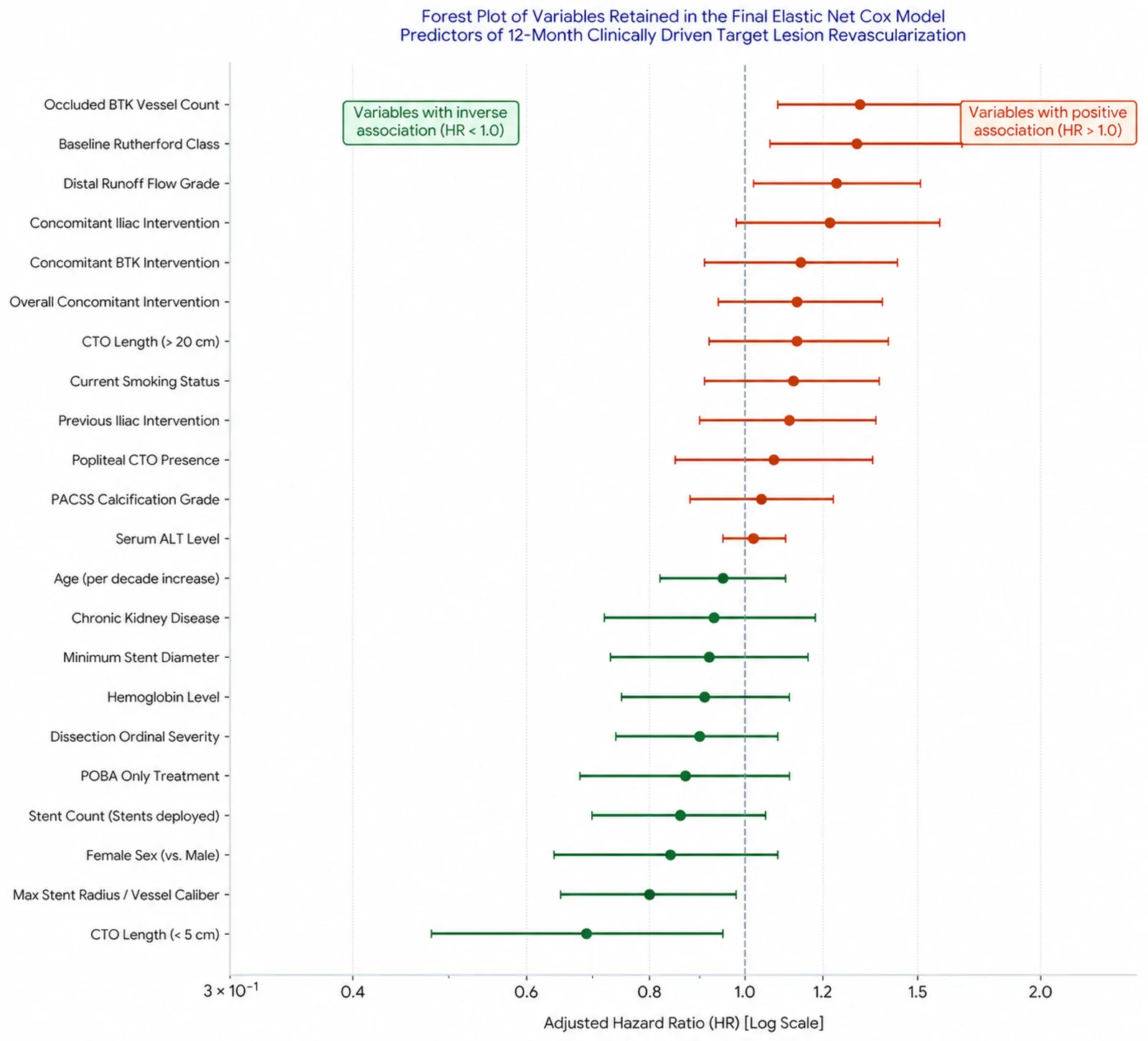
Forest Plot of Adjusted Hazard Ratios for Variables Retained in the Final Elastic Net Cox Model.

**Figure 4:**
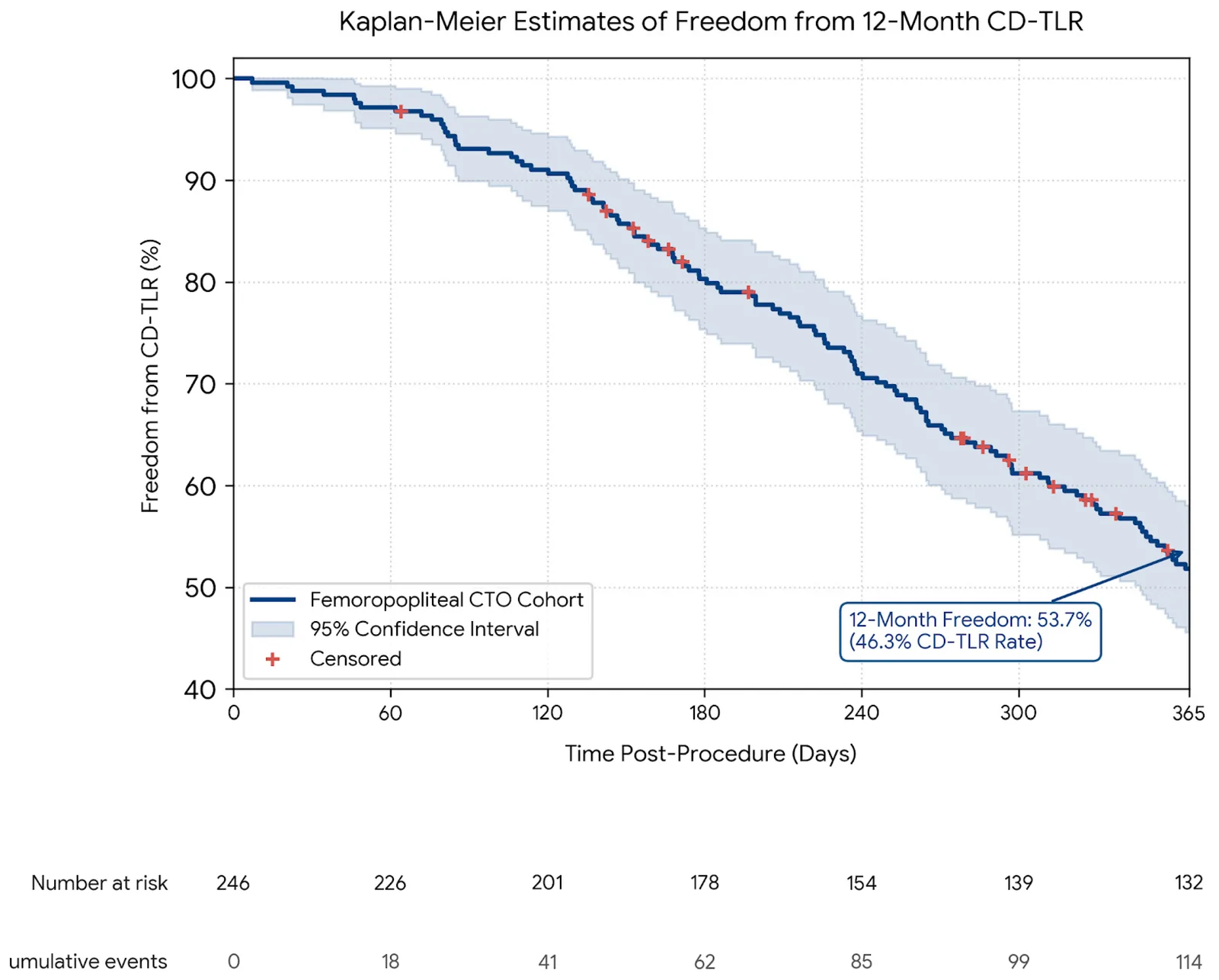
Kaplan–Meier Curve of Freedom from 12-Month Clinically Driven Target Lesion Revascularization (CD-TLR).

**Figure 5:**
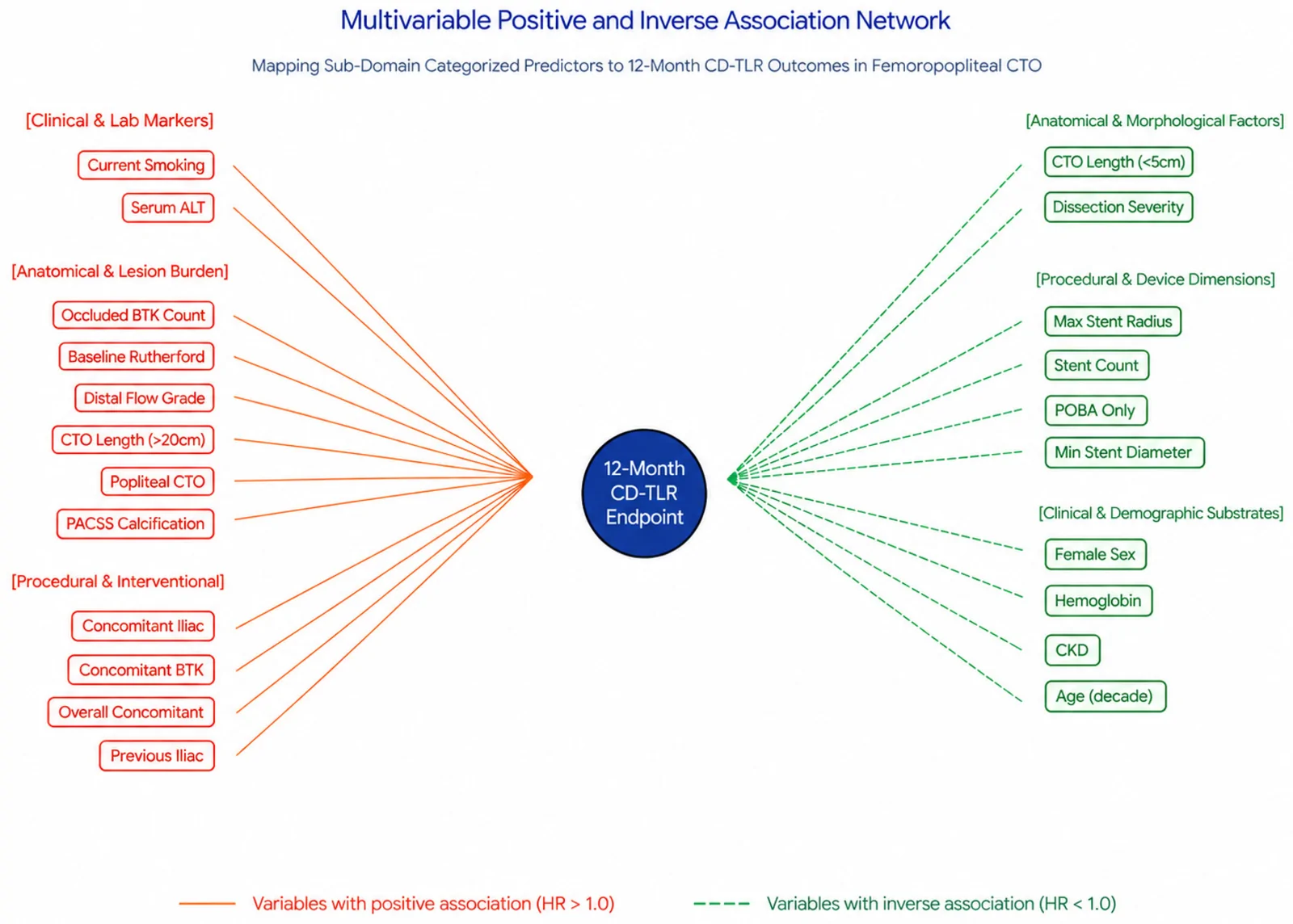
Multivariable Positive and Inverse Associations with 12-Month CD-TLR.

**Table 1:** Baseline Clinical, Anatomical and Procedural Characteristics of the Study Population According to 12-Month Clinically Driven Target Lesion Revascularization (CD-TLR) Status.

Variable	Overall Cohort (*n* = 246)	No CD-TLR Group (*n* = 132)	CD-TLR Group (*n* = 114)	*p*-Value
**Age (years)**	68.00 (61.00–74.00)	68.50 (61.00–73.25)	68.00 (61.00–74.00)	0.94
**Female Sex**	23 (9.3%)	10 (7.6%)	13 (11.4%)	0.42
**Serum Creatinine (mg/dL)**	0.96 (0.79–1.25)	0.97 (0.79–1.26)	0.94 (0.79–1.19)	0.54
**Fasting Blood Glucose (mg/dL)**	131.00 (99.25–189.50)	136.50 (99.75–188.50)	122.50 (99.50–190.50)	0.61
**Serum Albumin (g/dL)**	39.00 (33.00–42.75)	39.00 (33.00–42.00)	38.00 (33.25–43.00)	0.68
**Total Cholesterol (mg/dL)**	177.50 (144.00–212.00)	166.50 (142.00–211.00)	180.00 (150.00–215.50)	0.26
**LDL Cholesterol (mg/dL)**	103.00 (74.00–141.50)	94.50 (72.00–136.50)	112.50 (76.40–145.00)	0.08
**HDL Cholesterol (mg/dL)**	42.00 (35.25–47.92)	40.50 (34.00–46.00)	42.00 (37.00–49.75)	0.08
**Triglycerides (mg/dL)**	139.00 (104.00–194.75)	143.50 (111.50–192.25)	132.50 (102.00–202.00)	0.48
**White Blood Cell Count (10^3^/mL)**	8.59 (7.20–10.37)	8.35 (7.20–10.10)	8.70 (7.10–10.49)	0.39
**Hemoglobin (g/dL)**	12.95 (10.70–14.60)	12.90 (10.55–14.20)	13.05 (11.03–15.10)	0.08
**Platelet Count (10^3^/mL)**	241.50 (202.50–305.75)	240.50 (195.00–305.25)	243.50 (212.00–303.50)	0.48
**Hypertension**	152 (61.8%)	84 (63.6%)	68 (59.6%)	0.67
**Hyperlipidemia**	186 (75.6%)	96 (72.7%)	90 (78.9%)	0.33
**Diabetes Mellitus**	147 (59.8%)	81 (61.4%)	66 (57.9%)	0.67
**Chronic Kidney Disease**	71 (28.9%)	44 (33.3%)	27 (23.7%)	0.13
**Current Smoking Status**	131 (53.3%)	77 (58.3%)	54 (47.4%)	0.11
**Coronary Artery Disease**	172 (69.9%)	89 (67.4%)	83 (72.8%)	0.44
**Left Ventricular Ejection Fraction (%)**	65.00 (46.25–65.00)	65.00 (48.75–65.00)	65.00 (46.25–65.00)	0.68
**CHA_2_DS_2_-VASc**	4.00 (3.00–4.75)	4.00 (3.00–4.00)	4.00 (3.00–5.00)	0.48
**Previous PAD**	211 (85.8%)	116 (87.9%)	95 (83.3%)	0.50
**Previous Iliac Intervention**	59 (24.0%)	34 (25.8%)	25 (21.9%)	0.61
**Previous SFA Intervention**	214 (87.0%)	115 (87.1%)	99 (86.8%)	1.00
**Previous BTK Intervention**	113 (45.9%)	58 (43.9%)	55 (48.2%)	0.54
**Baseline Ankle-Brachial Index**	0.70 (0.70–0.80)	0.70 (0.60–0.80)	0.80 (0.70–0.80)	0.06
**Baseline Rutherford Class**	4.00 (4.00–5.00)	4.00 (4.00–5.00)	4.00 (4.00–4.00)	0.52
**CTOP Classification**				0.05
1	35 (14.2%)	12 (9.1%)	23 (20.2%)	
2	93 (37.8%)	57 (43.2%)	36 (31.6%)	
3	51 (20.7%)	26 (19.7%)	25 (21.9%)	
4	67 (27.2%)	37 (28.0%)	30 (26.3%)	
**Distal Runoff Flow Grade**				0.01
1	5 (2.0%)	3 (2.3%)	2 (1.8%)	
2	135 (54.9%)	84 (63.6%)	51 (44.7%)	
3	106 (43.1%)	45 (34.1%)	61 (53.5%)	
**TASC II Classification**				0.75
A	26 (10.6%)	13 (9.8%)	13 (11.4%)	
B	0 (0%)	0 (0%)	0 (0%)	
C	48 (19.5%)	24 (18.2%)	24 (21.1%)	
D	172 (69.9%)	95 (72.0%)	77 (67.5%)	
**Number of Occluded BTK Vessels**				0.02
0	46 (18.7%)	15 (11.4%)	31 (27.2%)	
1	84 (34.1%)	50 (37.9%)	34 (29.8%)	
2	87 (35.4%)	49 (37.1%)	38 (33.3%)	
3	29 (11.8%)	18 (13.6%)	11 (9.6%)	
**CTO Length < 5 cm**	26 (10.6%)	17 (12.9%)	9 (7.9%)	0.28
**CTO Length 5–10 cm**	23 (9.3%)	13 (9.8%)	10 (8.8%)	0.81
**CTO Length 10–20 cm**	38 (15.4%)	16 (12.1%)	22 (19.3%)	0.13
**CTO Length > 20 cm**	159 (64.6%)	86 (65.2%)	73 (64.0%)	0.86
**Popliteal CTO Presence**	30 (12.2%)	17 (12.9%)	13 (11.4%)	0.88
**Popliteal Trifurcation Involvement**	14 (5.7%)	10 (7.6%)	4 (3.5%)	0.27
**Total SFA Occlusion Length (>20 cm)**	161 (65.4%)	88 (66.7%)	73 (64.0%)	0.77
**PACSS Calcification Grade**				0.36
0	61 (24.8%)	27 (20.5%)	34 (29.8%)	
1	55 (22.4%)	34 (25.8%)	21 (18.4%)	
2	47 (19.1%)	25 (18.9%)	22 (19.3%)	
3	41 (16.7%)	21 (15.9%)	20 (17.5%)	
4	42 (17.1%)	25 (18.9%)	17 (14.9%)	
**Subintimal Crossing Technique**	180 (73.2%)	102 (77.3%)	78 (68.4%)	-
**Retrograde Approach Used**	53 (21.5%)	30 (22.7%)	23 (20.2%)	0.74
**Predilatation Balloon Diameter (mm)**				0.24
5	89 (36.2%)	38 (28.8%)	51 (44.7%)	
5–6	47 (19.1%)	23 (17.4%)	24 (21.1%)	
6	110 (44.7%)	71 (53.8%)	39 (34.2%)	
**Self-Expanding Stent Implantation**	112 (45.5%)	60 (45.5%)	52 (45.6%)	0.65
**Supera Stent Implantation**	66 (26.8%)	34 (25.8%)	32 (28.1%)	0.79
**Balloon-Expandable Covered Stent-Graft Implantation**	6 (2.4%)	5 (3.8%)	1 (0.9%)	0.28
**Stent Count (Number of Stents)**				0.40
0	86 (35.0%)	50 (37.9%)	36 (31.6%)	
1	86 (35.0%)	40 (30.3%)	46 (40.4%)	
2	56 (22.8%)	31 (23.5%)	25 (21.9%)	
3	18 (7.3%)	11 (8.3%)	7 (6.1%)	
**Plain Old Balloon Angioplasty (POBA) Only**	81 (32.9%)	47 (35.6%)	34 (29.8%)	0.41
**Total Stent Length (mm)**	100.00 (0.00–180.00)	100.00 (0.00–180.00)	100.00 (0.00–160.00)	0.83
**Post-Dilatation Performed**	26 (10.6%)	14 (10.6%)	12 (10.5%)	1.00
**Max Stent Diameter (mm)**	6.00 (0.00–6.00)	6.00 (0.00–6.00)	6.00 (0.00–6.75)	0.51
**Stent Overlap Presence**	70 (28.5%)	37 (28.0%)	33 (28.9%)	0.99
**Overall Concomitant Intervention**	78 (31.7%)	45 (34.1%)	33 (28.9%)	0.47
**Concomitant Iliac Intervention**	39 (15.9%)	23 (17.4%)	16 (14.0%)	0.58
**Concomitant BTK Intervention**	48 (19.5%)	27 (20.5%)	21 (18.4%)	0.81
**Dual Antiplatelet Therapy**	240 (97.6%)	130 (98.5%)	110 (96.5%)	0.55
**Dissection Severity Grade**				0.02
0	1 (0.4%)	1 (0.8%)	0 (0.0%)	
1	16 (6.5%)	3 (2.3%)	13 (11.4%)	
2	30 (12.2%)	13 (9.8%)	17 (14.9%)	
3	53 (21.5%)	33 (25.0%)	20 (17.5%)	
4	146 (59.3%)	82 (62.1%)	64 (56.1%)	
**Dissection Lumen Diameter Compromise**	147 (59.8%)	84 (63.6%)	63 (55.3%)	0.25
**Dissection Shape/Pattern**	195 (79.3%)	111 (84.1%)	84 (73.7%)	0.06
**Dissection Impaired Flow Grade**				0.78
0	21 (8.5%)	13 (9.8%)	8 (7.0%)	
1	173 (70.3%)	92 (69.7%)	81 (71.1%)	
2	44 (17.9%)	22 (16.7%)	22 (19.3%)	
3	8 (3.3%)	5 (3.8%)	3 (2.6%)	
**Dissection Morphology Grade**				0.38
0	5 (2.0%)	4 (3.0%)	1 (0.9%)	
1	152 (61.8%)	78 (59.1%)	74 (64.9%)	
2	89 (36.2%)	50 (37.9%)	39 (34.2%)	
**Post-Procedure ABI > 0.9**	240 (97.6%)	130 (98.5%)	110 (96.5%)	0.55

Data are presented as the **median (interquartile range [IQR])** for continuous variables and ***n* (%)** for categorical variables. Continuous variables were compared using the **Mann–Whitney U test**, and categorical variables were compared using the **Pearson χ^2^ test** or **Fisher’s exact test**, as appropriate. *p* values < 0.05 were considered statistically significant. **Abbreviations:** ABI, ankle–brachial index; ALT, alanine aminotransferase; AST, aspartate aminotransferase; BTK, below-the-knee; CAD, coronary artery disease; CHA_2_DS_2_-VASc, Congestive Heart Failure, Hypertension, Age, Diabetes, Stroke, Vascular Disease, Age, Sex category; CTO, chronic total occlusion; CTOP, chronic total occlusion crossing approach classification; HDL, high-density lipoprotein; LDL, low-density lipoprotein; PAD, peripheral artery disease; PACSS, Peripheral Academic Research Consortium calcium scoring system; POBA, plain old balloon angioplasty; SFA, superficial femoral artery; TASC, Trans-Atlantic Inter-Society Consensus.

**Table 2:** Twelve-Month Clinical and Reintervention Outcomes According to Clinically Driven Target Lesion Revascularization (CD-TLR) Status.

Follow-up Outcomes, *n* (%)	Overall Cohort (*n* = 246)	No CD-TLR Group (*n* = 132)	CD-TLR Group (*n* = 114)	*p*-Value
CD-TLR	114 (46.3%)	0 (0.0%)	114 (100.0%)	<0.001
—TLR via POBA Only	71 (28.9%)	0 (0.0%)	71 (62.3%)	<0.001
TLR via POBA + Stenting	43 (17.5%)	0 (0.0%)	43 (37.7%)	<0.001
Non-Target Lesion TVR (Other Vessel Segment)	2 (0.8%)	2 (1.5%)	0 (0.0%)	0.28
Major Amputation	2 (0.8%)	1 (0.8%)	1 (0.9%)	1.00
Surgical Bypass Surgery	2 (0.8%)	0 (0.0%)	2 (1.8%)	0.21
Catheter-Directed Thrombectomy	5 (2.0%)	2 (1.5%)	3 (2.6%)	0.67
In-Hospital Mortality	3 (1.2%)	1 (0.8%)	2 (1.8%)	0.60

Data are presented as ***n*** **(%)**. Categorical variables were compared using the **Pearson χ^2^ test** or **Fisher’s exact test**, as appropriate. *p* values < 0.05 were considered statistically significant. Both surgical bypass procedures and three of the five catheter-directed thrombectomy procedures involved the previously treated target lesion; these occurred in patients who had already undergone endovascular CD-TLR and were therefore not counted as separate CD-TLR events. The remaining two thrombectomy procedures involved non-target segments. **Abbreviations:** CD-TLR, clinically driven target lesion revascularization; TLR, target lesion revascularization; TVR, target vessel revascularization; POBA, plain old balloon angioplasty.

**Table 3:** Multivariable Elastic Net Cox Regression Model for 12-Month Clinically Driven Target Lesion Revascularization (CD-TLR).

Predictor Variable	Coefficient (β)	Adjusted Hazard Ratio (HR)	95% Confidence Interval
Variables With Positive Coefficients (β > 0)			
Occluded BTK Vessel Count	+0.2666	1.31	1.08–1.62
Baseline Rutherford Class	+0.2633	1.30	1.06–1.60
Distal Runoff Flow Grade	+0.2136	1.24	1.02–1.51
Concomitant Iliac Intervention	+0.1987	1.22	0.98–1.58
Concomitant BTK Intervention	+0.1315	1.14	0.91–1.43
Overall Concomitant Intervention	+0.1226	1.13	0.94–1.38
CTO Length (>20 cm)	+0.1217	1.13	0.92–1.40
Current Smoking Status	+0.1108	1.12	0.91–1.37
Previous Iliac Intervention	+0.1006	1.11	0.90–1.36
Popliteal CTO Presence	+0.0703	1.07	0.85–1.35
PACSS Calcification Grade	+0.0423	1.04	0.88–1.23
Serum ALT Level	+0.0210	1.02	0.95–1.10
Variables With Inverse Coefficients (β < 0)			
CTO Length (<5 cm)	−0.3707	0.69	0.48–0.95
Max Stent Radius/Vessel Caliber Marker *	−0.2282	0.80	0.65–0.98
Female Sex (vs. Male)	−0.1796	0.84	0.64–1.08
Stent Count (Number of Stents deployed)	−0.1544	0.86	0.70–1.05
Plain Old Balloon Angioplasty (POBA) Only	−0.1365	0.87	0.68–1.11
Dissection Ordinal Severity	−0.1109	0.90	0.74–1.08
Hemoglobin Level	−0.0945	0.91	0.75–1.11
Minimum Stent Diameter	−0.0820	0.92	0.73–1.16
Chronic Kidney Disease	−0.0773	0.93	0.72–1.18
Age (per decade increase)	−0.0512	0.95	0.82–1.10

Positive β coefficients (HR > 1.00) indicate positive associations with 12-month Clinically Driven Target Lesion Revascularization (CD-TLR), whereas negative β coefficients (HR < 1.00) indicate inverse associations. Hazard ratios are calculated as HR = exp(β). 95% Confidence Intervals were derived via 1000 bootstrap resamples. * Max Stent Radius is interpreted as a surrogate marker for native reference vessel caliber rather than an intrinsic protective effect of stent deployment (see Methods and Supplementary Table S3).

## Data Availability

The data that support the findings of this study are available from the corresponding author upon reasonable request. In accordance with international biomedical journal standards and ICMJE guidelines regarding data transparency for clinical research, the corresponding author will provide anonymized participant-level datasets, data dictionaries, and analytical R code upon reasonable request. Access to these materials will be granted to qualified researchers whose proposed analytical objectives align with the ethical approvals and institutional review board protocols governing this study. Requests for data sharing should be directed to the corresponding author via electronic mail and will be evaluated following institutional compliance reviews.

## Supplementary Materials

The following supporting information can be downloaded at https://www.mdpi.com/article/10.3390/jcm15197438/s1, Table S1: Unadjusted Univariable Cox Proportional Hazards Analysis of All 27 Candidate Predictors for 12-Month CD-TLR; Table S2: Discrimination and Predictive Performance Benchmarking of Survival Models for 12-Month Clinically Driven Target Lesion Revascularization (CD-TLR); Table S3: Univariable and Multivariable Cox Proportional Hazards Analysis of Stent Dimensions and Procedural Characteristics for 12-Month CD-TLR in Stented Patients (*n* = 160); Figure S1: Calibration Plot for the Final Elastic Net Cox Model Predicting 12-Month Clinically Driven Target Lesion Revascularization (CD-TLR). Additional methodological details regarding LASSO, Elastic Net, and Ridge regression are provided in the Supplementary Methods [9,23,24].
