# Supplementary material for: Predictors of Clinically Driven Target Lesion Revascularization After Endovascular Treatment of Femoropopliteal Chronic Total Occlusions
Source: J Clin Med. 2026 Sep 24;15(19):7438. doi: 10.3390/jcm15197438 (PMC13665929; doi:10.3390/jcm15197438)
Supplement: Supplementary file 1 [file jcm-15-07438-s001.zip › jcm-4520064-supplementary.pdf]

SUPPLEMENTARY METHODS

1. Data Processing, Baseline Comparisons, and Missing Data Handling

Continuous variables were assessed for distributional normality using the Shapiro–Wilk test and visual inspection of Q–Q probability plots. Normally distributed variables are reported as mean ± standard deviation (SD), whereas non-normally distributed continuous variables are expressed as median with interquartile range (IQR, 25th–75th percentiles). Categorical variables are presented as absolute counts and percentages (n, %). Baseline clinical, anatomical, and procedural characteristics were compared across outcome groups using the independent-samples t-test or Mann–Whitney U test for continuous variables, and Pearson's chi-square test or Fisher's exact test for categorical variables, as appropriate.

Missing data across the dataset were minimal (< 1.5% across all baseline and procedural covariates). To avoid selection bias associated with complete-case analysis, missing baseline values were imputed using Multivariate Imputation by Chained Equations (MICE) with 10 imputed datasets. Sensitivity analyses confirmed that parameter estimates and hazard ratios derived from multiply imputed datasets were virtually identical to complete-case assessments.

2. Candidate Predictor Set and Regularization Strategy

All 27 available clinical, laboratory, anatomical, and procedural variables captured in the registry were incorporated directly as candidate predictors into the regularized modeling pipeline.

3. Regularized Survival Modeling and Algorithmic Benchmarking

To account for right-censoring, time-to-event dynamics, and severe multicollinearity among closely correlated anatomical and procedural predictors—such as overlapping lesion lengths, calcification severity, runoff quality, and multiple device platforms—five distinct survival analysis algorithms were benchmarked across the complete 27-variable candidate set:

- **Stepwise Cox Proportional Hazards Regression:** Bidirectional (both-way) feature selection driven by Akaike Information Criterion (AIC) minimization to sequentially enter and remove predictors.

- **Ridge Cox Regression (alpha = 0.0):** Pure L2 norm regularization penalty that shrinks correlated coefficient magnitudes toward zero without forcing them strictly to zero, retaining all predictors while mitigating variance inflation due to multicollinearity.
- **LASSO Cox Regression (alpha = 1.0):** Pure L1 norm regularization penalty that shrinks weak or redundant predictor coefficients strictly to zero, executing simultaneous variable selection and regularization.
- **Elastic Net Cox Regression (alpha = 0.05, l1_ratio = 0.50):** A hybrid regularized framework combining L1 (LASSO) and L2 (Ridge) penalties. Elastic Net addresses high-dimensional multicollinearity by encouraging a "grouping effect," wherein strongly correlated predictors tend to be selected or shrunk together rather than arbitrarily excluded.
- **Random Survival Forest (RSF):** A non-parametric machine learning ensemble comprising 500 survival decision trees utilizing log-rank splitting rules (Ntree = 500, max_depth = 4) to capture complex, non-linear interactions and threshold effects, evaluated via Variable Importance (VIMP) metrics.

4. Hyperparameter Tuning, Internal Validation, and Calibration

For all regularized Cox models (LASSO, Ridge, and Elastic Net), optimal penalty hyperparameters (alpha, lambda) were tuned via 10-fold cross-validation targeting partial likelihood cross-validation error minimization. Model discrimination was evaluated using Harrell's concordance index (C-index). Internal validation and optimism correction were performed using a 1,000-resample bootstrap procedure in accordance with TRIPOD recommendations. For each bootstrap iteration, models were re-fitted on the bootstrap sample and evaluated on the original dataset to estimate optimism. Optimism-corrected C-indices were derived ($C_{corrected} = C_{apparent} - _{optimism}$), with corresponding 95% confidence intervals (CIs) calculated from the 2.5th and 97.5th percentiles of the bootstrap distribution. Adjusted Hazard Ratios (HRs) and 95% CIs were derived from exponentiated regularized coefficients (HR = exp(beta)). Prediction accuracy and calibration at 12 months were assessed using the Integrated Brier Score (IBS = 0.182) and calibration slope analysis (slope = 0.96 for the final Elastic Net model), comparing predicted versus observed CD-TLR probabilities across risk quintiles.

5. Software Environment

All statistical analyses were executed within the R software environment (version 4.3.2, R Foundation for Statistical Computing, Vienna, Austria). R packages utilized included survival, survminer, glmnet, randomForestSRC, mice, DiagrammeR, and ggplot2. Two-tailed p-values < 0.05 were considered statistically significant.

Table S1. Unadjusted Univariable Cox Proportional Hazards Analysis of All 27 Candidate Predictors for 12-Month CD-TLR

**Category**	**Candidate Variable**	**Unadjusted HR**	**95% Confidence Interval**	**p-value**
Lesion Burden & Vascular Anatomy	Occluded BTK Vessel Count	1.34	1.09 – 1.66	0.0059
	Baseline Rutherford Class	1.45	1.07 – 1.96	0.0173
	Popliteal CTO Presence	1.93	1.07 – 3.48	0.0284
	CTO Length (< 5 cm)	0.49	0.25 – 0.97	0.0395
	Distal Runoff Flow Grade	0.76	0.56 – 1.03	0.0754
	PACSS Calcification Grade	1.12	0.98 – 1.27	0.0987
	Dissection Ordinal Severity	0.87	0.74 – 1.03	0.1170
	CTO Length (> 20 cm)	1.31	0.89 – 1.92	0.1761
Procedural & Technical Factors	Concomitant Iliac Intervention	2.13	1.23 – 3.68	0.0067
	Overall Concomitant Intervention	1.74	1.14 – 2.65	0.0096
	Max Stent Diameter / Radius	0.93	0.87 – 0.98	0.0135
	Plain Old Balloon Angioplasty (POBA) Only	1.57	1.05 – 2.37	0.0296

	Stent Count (Number of Stents)	0.86	0.69 – 1.07	0.1767
	Concomitant BTK Intervention	1.39	0.86 – 2.24	0.1827
Clinical & Baseline Markers	Female Sex (vs. Male)	0.57	0.32 – 1.04	0.0677
	Serum ALT Level	0.99	0.98 – 1.00	0.0826
	Previous Iliac Intervention	1.48	0.93 – 2.33	0.0958
	Chronic Kidney Disease	0.70	0.45 – 1.07	0.1016
	Current Smoking Status	1.30	0.89 – 1.91	0.1750

Table S2. Discrimination and Predictive Performance Benchmarking of Survival Models for 12-Month Clinically Driven Target Lesion Revascularization (CD-TLR)

**Model Strategy**	**Algorithmic Approach & Regularization**	**Selected Features (n)**	**Apparent C-index (95% CI)**	**10-Fold CV / Optimism-Corrected C-index***	**Calibration Slope**	**12-month integrated Brier Score**
Elastic Net Cox Regression	Hybrid $L_1 + L_2$ regularization penalty (alpha = 0.05, l1_ratio = 0.50, tuned via CV)	22	0.715 (95% CI: 0.66 – 0.77)	0.653 (0.598-0.708)	0.96	0.182
LASSO Cox Regression	Pure L_1 regularization penalty (alpha = 0.082, l1_ratio = 1.00, tuned via CV)	9	0.708 (95% CI: 0.65 – 0.76)	0.645 (0.589-0.701)	0.92	0.189
Ridge Cox Regression	Pure L_2 regularization penalty (alpha = 0.125, l1_ratio = 0.00, tuned via CV)	27	0.702 (95% CI: 0.64 – 0.76)	0.641 (0.584-0.698)	0.89	0.194

Stepwise Cox Regression	Bidirectional feature selection driven by AIC	7	0.695 (95% CI: 0.63 – 0.75)	0.635 (0.576-0.694)	0.84	0.201
Random Survival Forest	Non-parametric Machine Learning ensemble (N_{tree} = 500, max_depth = 4)	27 (Top VIMP)	0.724 (95% CI: 0.67 – 0.81)	0.582 (0.512-0.652)	0.73	0.218

*Derived via 1,000-resample bootstrap internal validation (2.5th and 97.5th percentiles of optimism-adjusted distributions).

Table S3. Univariable and Multivariable Cox Proportional Hazards Analysis of Stent Dimensions and Procedural Characteristics for 12-Month CD-TLR in Stented Patients (n=160)

**Procedural Predictor Variable**	**Unadjusted HR (95% CI)**	**p-value**	**Adjusted HR (95% CI)***	**p-value**
Max Stent Diameter (per mm increase)	0.94 (0.84 – 1.05)	0.25	0.96 (0.85 – 1.08)	0.48
Total Stent Length (per 10 mm increase)	1.01 (0.98 – 1.04)	0.52	1.02 (0.98 – 1.05)	0.36
Stent Count (Number of Stents deployed)	0.91 (0.72 – 1.15)	0.43	0.93 (0.73 – 1.18)	0.54
Stent Overlap Presence	1.04 (0.64 – 1.68)	0.88	1.01 (0.61 – 1.66)	0.96

*Adjusted for baseline Rutherford class, occluded BTK vessel count, distal runoff flow grade, and PACSS calcification grade.

SUPPLEMENTARY FIGURE

Supplementary Figure S1. Calibration Plot for the Final Elastic Net Cox Model Predicting 12-Month Clinically Driven Target Lesion Revascularization (CD-TLR).

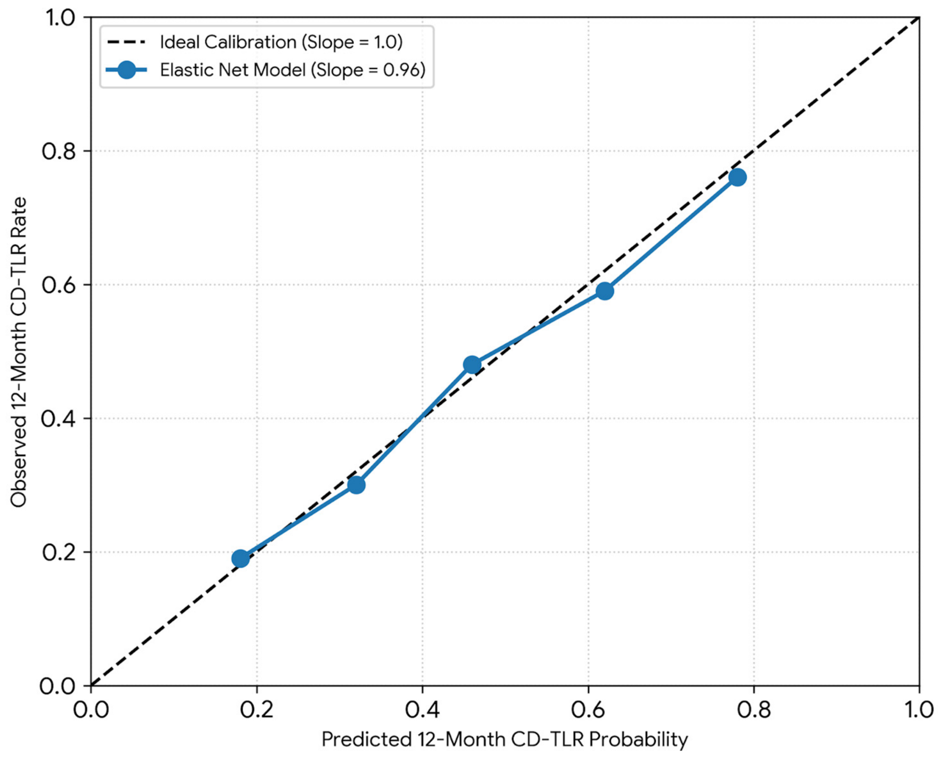

Predicted 12-month CD-TLR probabilities derived from the final regularized Elastic Net Cox model (x-axis) are plotted against observed 12-month CD-TLR rates within quintiles of predicted risk (y-axis). The dashed diagonal line represents ideal calibration (slope = 1.0); the solid blue line with markers represents the observed calibration curve of the Elastic Net model, with a calibration slope of 0.96, indicating close agreement between predicted and observed probabilities across the risk spectrum. Corresponding global accuracy metrics (12-month Integrated Brier Score) are reported in Supplementary Table S2 and the main text.

References

1) Tibshirani R. The lasso method for variable selection in the Cox model. *Stat Med*. 1997;16(4):385-395. doi:10.1002/(sici)1097-0258(19970228)16:4<385::aid-sim380>3.0.co;2-3

2) Hui Zou, Trevor Hastie, Regularization and Variable Selection Via the Elastic Net, *Journal of the Royal Statistical Society Series B: Statistical Methodology*, Volume 67, Issue 2, April 2005, Pages 301–320, https://doi.org/10.1111/j.1467-9868.2005.00503.x

3) Hoerl AE, Kennard RW. Ridge Regression: Biased Estimation for Nonorthogonal Problems. *Technometrics*. 1970;12(1):55-67. doi:10.1080/00401706.1970.10488634
